# Use of p38 MAPK Inhibitors for the Treatment of Werner Syndrome

**DOI:** 10.3390/ph3061842

**Published:** 2010-06-04

**Authors:** Mark C. Bagley, Terence Davis, Paola G. S. Murziani, Caroline S. Widdowson, David Kipling

**Affiliations:** 1 School of Chemistry, Main Building, Cardiff University, Park Place, Cardiff, CF10 3AT, UK; 2 Department of Pathology, School of Medicine, Cardiff University, Heath Park, Cardiff, CF14 4XN, UK

**Keywords:** accelerated ageing, inflammation, microwave-assisted synthesis, p38 MAPK, progeroid syndrome, Werner syndrome

## Abstract

Werner syndrome provides a convincing model for aspects of the normal ageing phenotype and may provide a suitable model for therapeutic interventions designed to combat the ageing process. Cultured primary fibroblast cells from Werner syndrome patients provide a powerful model system to study the link between replicative senescence *in vitro* and *in vivo* pathophysiology. Genome instability, together with an increased pro-oxidant state, and frequent replication fork stalling, all provide plausible triggers for intracellular stress in Werner syndrome cells, and implicates p38 MAPK signaling in their shortened replicative lifespan. A number of different p38 MAPK inhibitor chemotypes have been prepared rapidly and efficiently using microwave heating techniques for biological study in Werner syndrome cells, including SB203580, VX-745, RO3201195, UR-13756 and BIRB 796, and their selectivity and potency evaluated in this cellular context. Werner syndrome fibroblasts treated with a p38 MAPK inhibitor reveal an unexpected reversal of the accelerated ageing phenotype. Thus the study of p38 inhibition and its effect upon Werner pathophysiology is likely to provide new revelations into the biological mechanisms operating in cellular senescence and human ageing in the future.

## 1. Premature Ageing Disorders and Werner Syndrome

Ageing is the universal, progressive, and intrinsic accumulation of deleterious changes that compromise the physiological capability of an organism and ultimately lead to death. In recent years it has become clear that fundamental basic mechanisms of ageing exist that are conserved through evolution in species as diverse as *Drosophilia*, *C. elegans*, mice and humans, and which contribute to age-related tissue degeneration [1]. Understanding, and by extension intervening, in these processes offers a novel approach to understand and treat age-related diseases and degenerations, and represents an exciting new stage in ageing research.

Despite these potential benefits, several practical difficulties underlie the study of human ageing, most importantly the polygenic nature of many of the associated pathologies. An alternative to the study of whole body ageing in normal humans is the study of human monogenetic syndromes whose phenotypes show specific characteristics of ageing, so-called progeroid syndromes. Such syndromes are studied not only because of their severity for the patient, leading in many cases to lifespan shortening, but also in the expectation that their study may identify causative genes involved in the mechanisms underlying normal ageing [2,3]. 

To date, most progeroid syndromes for which genes and patho-physiological mechanisms have been identified are monogenic and classified as segmental, insofar as they are associated with many, but not all, of the clinical characteristics seen in normal ageing processes [4,5]. The validity of progeroid syndromes as ageing models is disputed, however, as they are not simply a global acceleration of age-linked pathology [6]. Several of the age-related symptoms are more or less severe than seen in normal ageing, and not all tissues show symptoms of premature senescence. In addition, progeroid syndromes are associated with non-age-related symptoms and are thus not fully reflective of the normal ageing process. However, in those aspects where premature ageing occurs, the process and pathology are remarkably similar to that seen in normally aged individuals. Indeed, it is the segmental nature of these syndromes that makes them tractable to analysis [1].

Werner syndrome (WS) is one of the better-characterized premature ageing disorders. Patients with WS have a large number of signs and symptoms of ageing that manifest at a younger age than normal. There are several other premature ageing syndromes where the patients display features of normal ageing at an early age, including Cockayne syndrome, Rothmund Thomson and Hutchinson Gilford progerias, which are all very rare conditions in the general population [7].

### 1.1. Pathophysiology of Werner Syndrome

Werner syndrome is an autosomal recessive human genetic instability and cancer predisposition syndrome with features of premature ageing, first characterized by Dr Otto Werner [8]. It affects approximately ten per million individuals [9]. The first sign of this disorder is often the lack of the pubertal growth spurt and WS individuals are typically short in height [1,5]. WS is characterized by juvenile bilateral cataracts, skin atrophy and sclerosis, premature hair-greying, and thymic atrophy. WS individuals appear much older than their chronological age; the premature ageing phenotypes associated with WS usually manifest themselves in the twenties or thirties, with the median age of diagnosis being the early thirties. WS individuals show increased predisposition to age-related diseases such as type II diabetes mellitus, osteoporosis, and atherosclerosis. Several of the clinical features are much more severe than seen in normal ageing, including calcification of the cardiac valves, atrophy of the testicles and skin appendages, scleroderma-like skin, and ulcerative lesions that develop over pressure points. WS individuals do not have any obvious immune system dysfunction despite the thymic atrophy, and they lack notable pathology of the central nervous system [10], although there are some reports of premature senile dementia [11]. Death generally occurs in the fourth or fifth decade of life, primarily from cancer or cardiovascular disease. 

An elevated incidence of cancer is observed in WS, although this is generally limited to rare non-epithelial cancers, especially mesenchymal cancers such as sarcomas [12,13]. Five neoplasms predominate in the disease: soft tissue sarcomas, thyroid carcinoma, osteosarcoma, and lentigenous melanoma and meningioma [12,14].

Overall, WS provides a convincing model for aspects of the normal ageing phenotype and may provide a suitable model for future potential therapeutic interventions designed to combat the ageing process [5,15].

### 1.2. The WS Cellular Phenotype

Cultured cells from normal individuals have a finite capacity to divide, after which they enter a state of permanent cell-cycle arrest termed ‘replicative senescence’. Cellular senescence has been postulated to contribute to normal human ageing and shows acceleration in WS [1,5]. Primary fibroblasts from WS patients show a very abbreviated *in vitro* lifespan, they divide more slowly than normal cells, exit the cell cycle at a higher rate than normal fibroblasts and so senesce more rapidly [16]. This reduced replicative cellular lifespan is not seen in all cell types, as T cells from WS individuals do not show premature senescence in culture [17], and interestingly there is no immune dysfunction reported in WS. This correlation between the different *in vitro* behaviour of various cell types and the *in vivo* ageing of the tissues from which these cells are derived, has led to the hypothesis that WS may be caused by accelerated senescence of a subset of division competent cells [18]. Thus cultured primary fibroblast cells from WS patients may provide a powerful model system to study the link between replicative senescence *in vitro* and normal ageing *in vivo* [1]. However, as the pathways leading to replicative senescence appear to be conserved in old WS fibroblasts, the actual process of early senescence is not yet fully understood [19,20].

### 1.3. The Werner Protein (WRNp) and Genome Instability in WS

Since the cloning of the gene responsible for WS in 1996 [21], the development of new experimental techniques has helped to define the molecular pathology of WS. Several of these approaches have focused on defining the cellular role of the Werner protein, WRNp, encoded by the gene defective in the disease, *WRN*. The WRNp is a nuclear 1,432-amino acid protein [21]. It belongs to the human RecQ helicase protein family and has a 3′→5′ helicase domain, but uniquely is known to include a 3′→5′ exonuclease domain which processes from the end of a DNA strand. The WRNp is found predominantly in the nucleoli but relocates to replication foci at the S-phase of the cell cycle, and to sites of DNA damage in response to DNA-damaging agents such as 4-nitroquinoline-1-oxide [22,23,24]. 

The WRNp interacts with numerous proteins involved in DNA metabolism and processing, including replication protein A, p53, proliferating cell nuclear antigen, topoisomerase I, DNA polymerase δ, and the Ku complex [25,26], suggesting that WRNp plays a role in DNA replication, recombination and repair processes. A number of studies have indicated that WRNp can unwind and/or hydrolyse a number of distinctive DNA structures, such as duplex DNA, branched DNA structures, D-loops, tetraplex DNA, four-way DNA junctions and long tracts of telomeric DNA [27,28,29,30,31]. Many of these structures may form during, and thus impair, processes of DNA metabolism. In addition, WRNp can drive branch migration of Holliday junctions and thus might accelerate and facilitate DNA recombination [32]. In addition, WRNp interacts with TRF1/TRF2 and POT1, proteins essential for the integrity of telomeres [33], and telomere defects have been reported in WS cells [34,35,36,37]. Finally, WRNp stabilizes DNA fragile sites acting in a common pathway with the cell cycle checkpoint protein ATR [38]. 

These data suggest a role for WRNp in the successful transit of certain DNA sequences through replication forks during DNA synthesis, e.g. telomeres and fragile sites, and primary WS cells display genome instability, manifested by telomere dysfunction, an elevated rate of chromosomal translocation and genomic deletions (particularly at fragile sites) [35,38,39], and are hypersensitive to a subset of DNA damaging agents [40], suggesting that the WRNp plays a key role in the cellular response to specific types of DNA damage. These features have led to WS being classified as a genome instability syndrome [41]. 

### 1.4. Telomere-Dependent and Telomere-Independent Mechanisms of Cell Ageing in WS Cells

Most normal human somatic cells are capable of only a finite number of divisions, after which they enter a state of viable growth arrest, so-called replicative senescence. Cellular senescence in normal human fibroblasts appears to be triggered by telomere erosion [42,43,44,45]. Recent studies suggest WRNp participates in pathways at telomeric ends and WS cells display some defects in telomere metabolism, including increased apparent rates of telomere shortening [34] (although this has been argued to be an artifact caused by an altered growth dynamics of WS cells [46]), and deficiencies in repair at telomeres [47]. In addition, young WS fibroblasts have telomeres of similar length to young normal cells, but senesce with longer mean telomere lengths than normal fibroblasts [34], perhaps suggesting that WS cells are more sensitive to variations in telomere length. 

However, although several groups have shown that telomere shortening acts as a primary driver of senescence in WS fibroblasts [48,49,50], it has been shown that this telomere-driven senescence synergizes with an additional telomere-independent mechanism in WS, indicating that telomere dynamics in WS fibroblasts are not significantly different from those in normal fibroblasts [46]. These data would therefore suggest that the accelerated replicative decline seen in WS fibroblasts does not arise from accelerated telomere erosion, but rather from a telomere-independent senescence mechanism. This telomere-independent senescence mechanism could be related to extrinsic stimuli, such as oxidative stress, or intrinsic signals such as genome instability or DNA damage. This telomere-independent senescence mechanism would be superimposed upon the normal telomere driven-dependent replicative arrest and thus the two mechanisms, working in concert, act to define the maximum possible lifespan of a WS culture [51].

**Figure 1:**
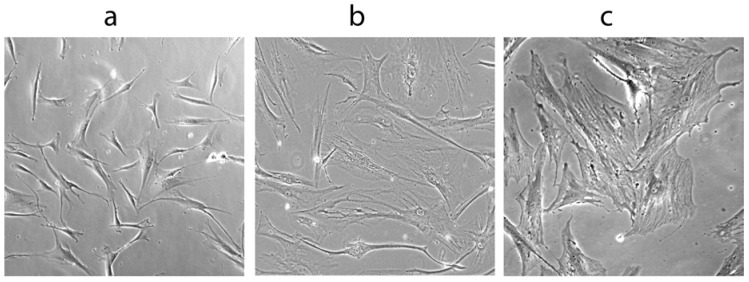
(a) Normal human fibroblasts. (b) Human fibroblasts showing a senescent morphology. (c) Young Werner syndrome cells.

Several characteristics of WS cells, such as very slow growth rates, an elongated cell cycle S-phase, and a morphology resembling aged normal fibroblasts even at low passages [52,53,54], suggest that WS cell ageing is not simply accelerated normal cell aging. These features are reminiscent of cells grown under conditions of stress [55,56,57,58], and many young WS cells resemble fibroblasts that have undergone oncogenic ras- or arsenite-induced premature senescence (Figure 1). Thus, WS cells show many of the characteristics of cells growing under ‘replication stress’ [59]. Exposure to DNA damaging agents as ‘stress signals’, has been coupled with a significantly elevated frequency of deletional mutations *in vitro* and defines a mutator phenotype [39]. The genome instability seen in WS, together with the frequent DNA replication fork stalling [60], provides a plausible trigger for the replication stress in WS cells. These changes in cell signaling are candidates for inducing a shortened replicative life span and forming the basis of a telomere-independent “stress-induced” premature senescence of WS cells.

### 1.5. Werner Syndrome and Inflammation

The pro-inflammatory cytokines such as tumor necrosis factor-α (TNF-α) and interleukin-1β (IL-1β) mediate the inflammatory response associated with the immunological recognition of infectious agents. The central role of pro-inflammatory cytokines in inflammatory disease processes, such as rheumatoid arthritis, osteoarthritis, psoriasis and inflammatory bowel disease has been established through evidence associating various cytokines with these diseases and through successful therapeutic intervention targeting these inflammatory molecules [61,62,63,64,65,66]. Normally the cytokines help regulate the body’s response to infections and cellular stresses [67]. Excessive production of TNF-α and IL-1β is believed to underlie the progression of many inflammatory diseases [64]. In recent years significant progress has been made in the identification of the signal transduction mechanisms responsible for inducing inflammatory gene expression that are fundamental in the initiation of the inflammatory response. Products of induced inflammatory genes include cytokines, chemokines and adhesion molecules (ICAM-1) that serve to promote recruitment of immunocompetent cells from the circulation to the affected site from an inflammatory injury. In recent years, substantial efforts have been made to define the intracellular signaling cascade in cells that mediate inflammatory processes. The mitogen activated protein kinase (MAPK) superfamily constitutes major hubs of this intracellular signaling due to their consistent activation by pro-inflammatory cytokines, and their role in nuclear signaling. The members of this family of kinases have come to be appreciated as key cellular signal transducers and attractive targets for drug development [68]. 

Fibroblasts derived from individuals with WS display activation of inflammatory signaling pathways and WS individuals exhibit high plasma levels of inflammatory cytokines [51,69] and ICAM-1 [70]. In addition, WS individuals have elevated levels of inflammatory diseases [5]. Thus Werner syndrome could be classified as an example of a class of diseases known as ‘inflamm-ageing’ [71,72] to underline the known association between inflammatory changes and many age-associated disease pathologies [71,73]. 

## 2. Mitogen Activated Protein Kinase Signal Transduction

**Figure 2:**
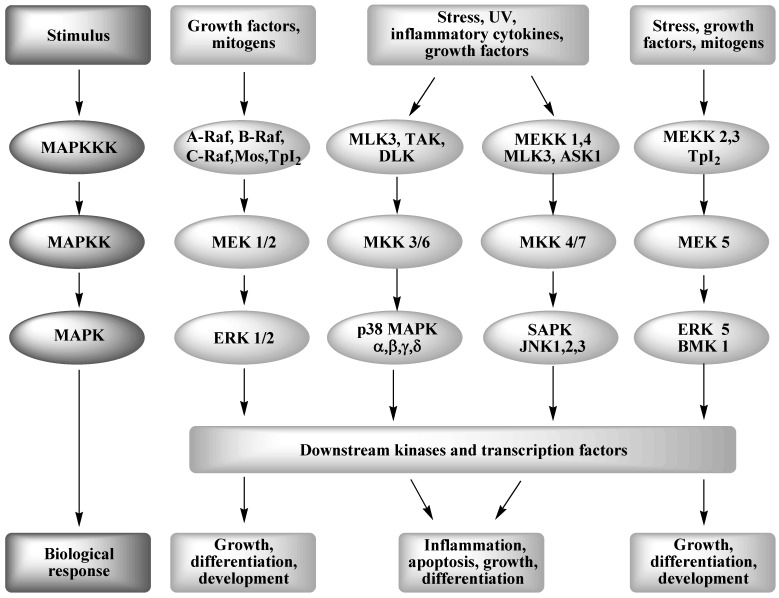
Mitogen activated protein kinase signaling cascades.

MAP kinases are intracellular signal transduction molecules, a group of protein serine/threonine and tyrosine kinases that are activated in response to a variety of extracellular stimuli and mediate signal transduction from the cell surface to the nucleus, and also modulate the function of many cytoplasmic proteins and processes (reviewed in [74]). The MAPK superfamily consists of at least four broad families, namely extracellular-signal-regulated kinase (ERK), Jun-NH2-terminal kinases (JNKs), ERK5 (or big MAPK1, BMK-1) and p38 MAPK. MAP kinases, in combination with several other signaling pathways, can differentially alter the phosphorylation status of transcription factors in a pattern unique to a given external signal. Classical MAP kinase signaling occurs in cascades, involving MAP kinases (MAPK) that are activated by MAPK kinases (MAPKK), which are in turn activated by MAPKK kinases (MAPKKK). The activation of specific MAPKK kinases through extracellular signals, such as endotoxins, stress, inflammatory cytokines TNF-α and IL-1β and growth factors, leads to phosphorylation and activation of downstream MAPK kinases that, in turn, phosphorylate and activate their MAP kinase. The activated MAP kinases can activate downstream kinases and transcription factors leading to changes in mRNA steady-state levels for messages that encode cytokines and receptors (amongst others). The MAPKs regulate several physiological and pathological cellular phenomena, including, cell cycle progression, cell proliferation and differentiation, apoptotic cell death, oncogenic transformation, and tumor cell invasion and metastasis, and are involved in the processes of inflammation and immunity (Figure 2). 

### 2.1. The JNK (SAPK) Pathway

The JNK MAPKs, also known as stress-activated MAPKs [74], were initially discovered by their ability to phosphorylate the proto-oncogene c-Jun [75]. The JNKs are encoded by three genes, JNK1, JNK2, and JNK3, that produce at least 10 protein subtypes [76]. The JNK1/2 subtypes are ubiquitously expressed, whereas the JNK3s are restricted mainly to testis and brain. The JNKs are characterized by bis-phosphorylation on a Thr-Gly-Tyr motif located in a region known as the activation loop by the dual-specificity serine/threonine MAPK kinases MKK4 and MKK7. The JNKs play major roles in apoptosis, proliferation, differentiation and inflammation. In addition to c-Jun, downstream targets include several transciption factors and protein kinases and the tumour suppressive protein p53. 

### 2.2. The ERK Pathway

ERK1/2, also known as p44^MAPK^ and p42^MAPK^, respectively, were the first MAPKs to be described in mammals [77]. They are widely expressed in mammalian tissues and are activated by growth factors and mitogens and play roles in cell proliferation, differentiation and survival [75]. The activation of ERK1/2 induces proliferative signals that may contribute to normal and cancerous growth [78,79]. Consistently, aberrant activation of the ERK pathway is a frequent event in human carcinogenesis. Upstream kinases are the MEK1/2 MAPK kinases and Raf MAPK kinase kinases (Figure 2). ERKs phosphorylate and stimulate the activities of many nuclear transcription factors, especially those involved in cellular proliferation, e.g. c-Fos, c-Jun and c-Myc [75]. 

### 2.3. The p38 MAPK Pathway

**Figure 3:**
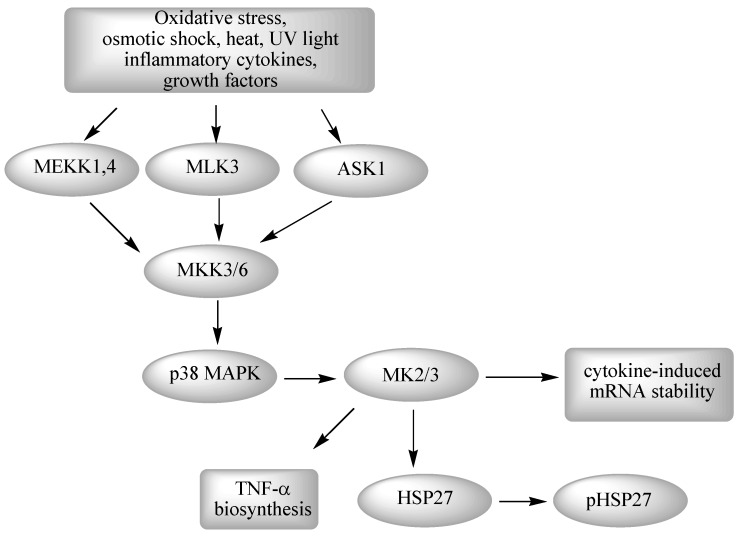
The p38 MAPK signaling pathway.

It has been established that the p38 MAP kinase pathway is activated in response to physical stress signals such as osmotic shock, heat, UV light and in response to pro-inflammatory cytokines such as TNF-α, IL-1 and growth factors. The p38 kinase is widely expressed in many cell types, including immune, inflammatory and endothelial cells. There are four p38 subtypes (α, β, γ and δ), each encoded by a separate gene, with the p38α kinase being believed to be the family member primarily responsible for regulation of inflammation [80,81]. P38α MAPK is activated through Thr-Gly-Tyr bis-phosphorylation in the activation loop, leading to increased production of pro-inflammatory cytokines such as TNF-α and IL-1β. Activation is achieved by dual-specificity serine/threonine MAPK kinases, MKK3 and MKK6, which are the upstream MAPK kinases responsible for p38 activation. A major primary substrate of p38 MAPK is MAPK activated protein kinase 2 (MAPKAPK-2 or MK2) [82] which in turn phosphorylates the small heat shock protein HSP27. This protein promotes polymerization of actin filaments and maintains integrity of the cytoskeleton (Figure 3) [56]. The p38α MAP kinase is a serine-threonine kinase that plays a central role in numerous pro-inflammatory responses. An important and accepted therapeutic approach for potential drug intervention in inflammatory disease is the reduction of pro-inflammatory cytokines [83].

### 2.4. WS and MAPK signaling

Stress-induced premature cellular senescence induced by oncogenic ras or oxidative stress can result from activation of the stress-associated p38 mitogen-activated protein kinase (MAPK) via the activating MAP kinase kinase 6 (MKK6) [57,58], and the use of the p38 selective inhibitor SB203580 prevents ras-induced senescence in human BJ fibroblasts [58]. Activation of p38 leads to the stabilization of the cyclin-dependent kinase inhibitor p21^WAF1^ and subsequent cell-cycle arrest [58,84]. The similarities between young WS cells and normal cells that have undergone ras-induced premature senescence raise the possibility that the p38 pathway may play a role in the premature senescence seen in WS cells.

The genome instability seen in WS, together with their increased pro-oxidant state [85,86], and frequent replication fork stalling, all provide plausible triggers for intracellular stress in WS cells, and thus implicate stress-induced MAPK signaling (including p38 activation) in inducing the shortened replicative lifespan. Indeed, p38 has been shown to be activated in young WS cells compared to young normal cells, together with phosphorylation of MK2 and HSP27 [87,88]. It is intriguing that p38 (and MK2) has been implicated in cardiovascular disease, diabetes, and osteoporosis, all of which are inflammatory features of WS. If premature aging of cells and p38 activation do underlie the accelerated ageing seen in WS individuals, these observations provide a rationale for a study of p38 MAPK inhibitors in the cellular physiology of WS. 

As different cells types in WS cells behave differently with respect to growth and cellular lifespan, and that this correlates with the *in vivo* ageing of the tissues from which these cells are derived, cultured primary fibroblast cells from WS patients may provide a powerful model system to study the link between replicative senescence *in vitro* and normal ageing *in vivo* [1]. However, it must be noted that this is only one tissue type and it is possible that the results may not translate into a full understanding of WS cellular biology, as p38 may well have differential effects in different cell types.

In contrast to p38, no activation of ERK1/2 is observed in WS cells above that seen in normal cells, and only limited JNK1/2 activation has been found in WS cells compared to normal cells (our unpublished observations). Initial studies using c-Raf inhibitors (the ERK1/2 upstream MAPK kinase kinase) showed no effects upon WS fibroblasts [89] and the JNK inhibitor SP600125 resulted in growth arrest (our unpublished observations). 

## 3. Use of MAPK Inhibitors for the Treatment of Werner Syndrome

**Figure 4:**
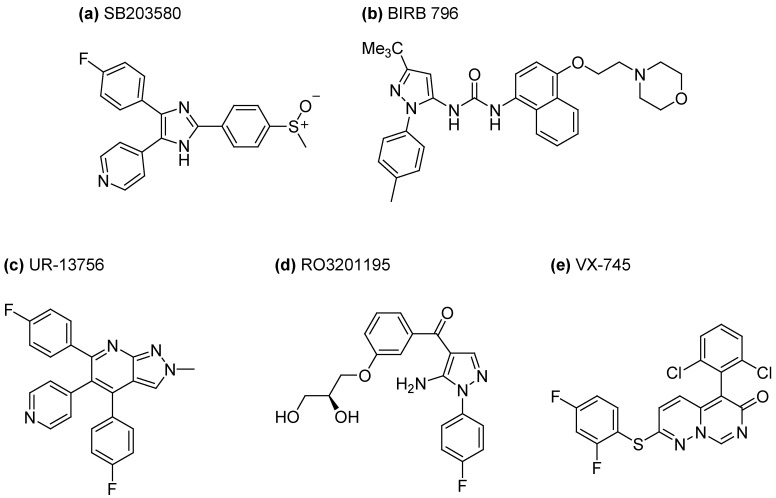
P38 inhibitor chemotypes studied in WS cells. (a) The prototypical inhibitor SB203580. (b) The Boehringer-Ingelheim inhibitor BIRB 796. (c) The Palau Pharma inhibitor UR-13756. (d) The Roche inhibitor RO3201195. (e) The Vertex inhibitor VX-745.

The discovery and development of selective, efficacious and safe small-molecule p38 mitogen activated protein kinase inhibitors for the treatment of inflammatory diseases remains the focus of numerous pharmaceutical research programs because of its crucial role in regulating the production of pro-inflammatory cytokines [90]. Blocking this kinase cascade may offer an effective therapy for treating many inflammatory diseases. The success of novel treatments has demonstrated the clinical benefit that can be gained from therapeutic intervention in cytokine signaling, highlighting the importance of pro-inflammatory cytokine systems like IL-1β and TNF-α as validated targets [91]. A number of molecular targets have been identified for the development of such small molecular agents but p38 mitogen activated protein (MAP) kinase is a major target because it occupies a central role in the regulation of IL-1β and TNF-α signaling networks at both the transcriptional and translational level [80,83]. In 1994 it was discovered that p38 was the molecular target of a novel class of cytokine suppressive inhibitors, exemplified by the prototypical inhibitor, pyridinylimidazole SB203580 (Figure 4a) [92]. These prototypes were originally prepared as inflammatory cytokine synthesis inhibitors that were subsequently found to be selective inhibitors of p38 MAP kinase. SB203580 inhibits the catalytic activity of p38 MAP kinase by competitive binding in the ATP pocket.

The development of therapeutic agents targeting p38 MAPK signaling has until recently focused upon small molecule ATP-competitive and also ATP-noncompetitive inhibitors of p38 MAPK. Structural knowledge of the binding site and the interactions made by ATP (**1**) is therefore a key factor in the understanding of how potent, selective compounds can be developed for such a structurally conserved family of enzymes. Many inhibitors have been designed to take advantage of specific interactions that are located within or near the ATP (**1**) binding site of p38. One feature that has proven to be important in terms of p38 inhibitor design, and kinase inhibitor design in general, has been the ability of the inhibitor to imitate ATP (**1**) and occupy regions within the ATP-binding cleft and therefore to mimic specific important H-bond interactions that occur in the ATP–kinase complex, while taking advantage of additional binding regions that are not utilized by ATP (**1**).

**Figure 5:**
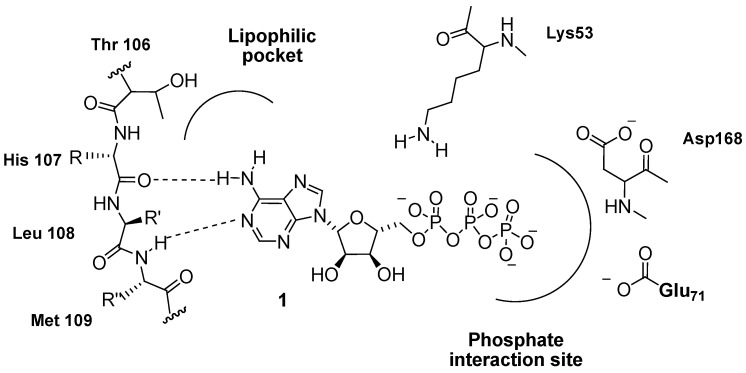
ATP binding modes with p38α MAPK.

Previous evidence from X-ray crystallographic structures of other kinases co-complexed with ATP analogues [93,94] suggest that bound ATP (**1**) directly interacts with the p38 hinge region residues His107 and Met109 to form a pair of hydrogen bonds with the adenine ring of ATP (**1**) (Figure 5). 

In addition highly conserved residues, such as Lys53, Glu71 and Asp168, are most likely involved in the coordination of the triphosphate group within the phosphate-binding region. Orientation of the triphosphate group in this manner allows for efficient catalysis of the γ-phosphorylation transfer to the substrate protein upon p38 activation [95]. 

**Figure 6:**
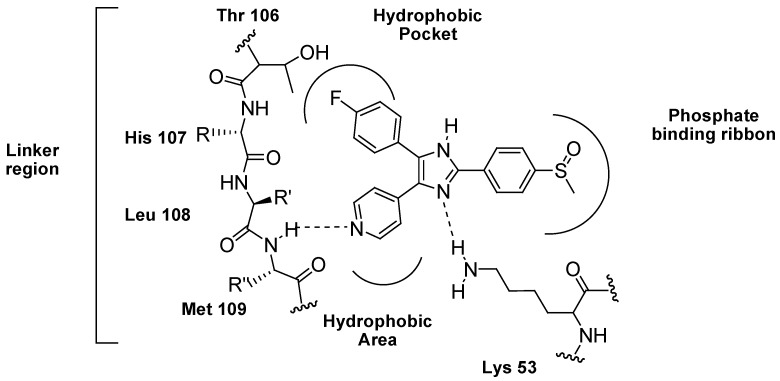
SB203580 binding modes with p38α MAPK.

Studies of p38 MAP kinase have shown that this enzyme possesses a hydrophobic pocket not occupied by ATP (**1**). This provides opportunities for the discovery and design of selective small molecule ATP-competitive inhibitors that imitate the ATP adenine and gain selectivity by occupying areas unfilled by ATP (**1**). Crystallographic mutational and biochemical studies by researchers of Boehringer Ingelheim demonstrated that SB203580 and related pyridin-4-yl imidazole derivatives bind in the ATP binding site of p38. The pyridine ring nitrogen of the inhibitor forms a hydrogen bond to the backbone NH of Met109 in the linker region. This is a common feature observed in all crystal structures of p38 in complex with respective pyridin-4-yl imidazole derivatives. This H-bond underlines the crucial importance of the pyridine ring for biological activity [96,97,98]. While the 4-fluorophenyl ring of SB203580 binds to a hydrophobic region which in p38 is represented by an unusually spacious pocket; a second hydrophobic area below the linker region is left unoccupied by simple pyridin-4-yl imidazole inhibitors (Figure 6) [95,97].

Numerous efforts have been made to discover structurally-related p38 inhibitors that maintain, or, in some cases, improve potency but also minimize liabilities that have been identified in the original pyridine-imidazole series. A multitude of monocyclic and fused heterocycles have been employed as scaffolds for the essential pyridin-4-yl-4-fluorophenyl pharmacophore with the aim to increase the selectivity (IC_50_ 39.5 nM) [90,99].

### 3.1. Inhibition of P38 MAPK Signaling in WS Cells by the Prototypical Inhibitor SB203580

**Figure 7:**
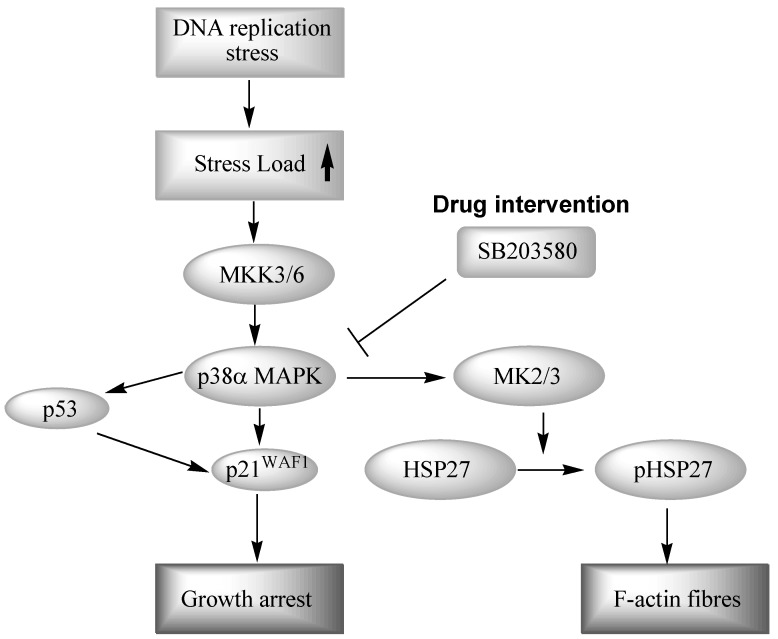
Drug intervention in premature growth arrest in WS cells.

The genome instability seen in WS, together with the frequent replication fork stalling and increased pro-oxidant state, provides a plausible trigger for replication stress in WS and young WS cells resemble fibroblasts that have undergone stress-induced premature senescence [88]. As stress-induced premature senescence due to several stimuli is known be transduced by p38 [55,57,84] this implicates the involvement of p38 signaling in the premature cell cycle arrest and the short replicative life span (Figure 7). P38 MAPK is indeed activated in young WS cells with associated high levels of the cyclin-dependent kinase inhibitor p21^WAF1^ and phosphorylated HSP27 [88]. Activation of p38 also leads to the production of F-actin stress fibres that result in the altered morphology that is typical of senescent cells [55].

**Figure 8:**
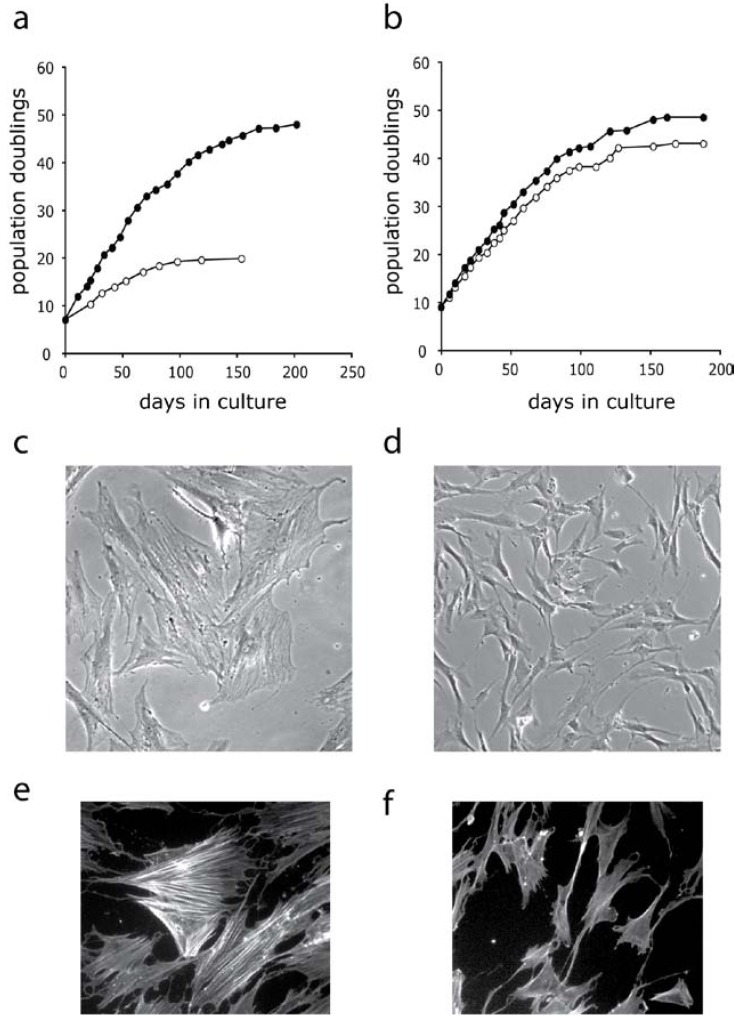
Effects of SB203580 on the growth rate and lifespan on WS fibroblasts (a) and normal dermal fibroblasts (b): SB203580 treated cells are closed circles; untreated cells are open circles. Effects on cellular morphology and stress fibre phenotype of WS fibroblasts: (c, e) untreated, (d, f) SB203580 treated.

The role of p38-transduced stress signaling in Werner syndrome cells has been investigated using SB203580, the cytokine-suppressive anti-inflammatory drug whose major inhibitory target is p38. Drug treatment reveals an unexpected reversal of the ageing phenotype of WS fibroblasts, which show a much-increased replicative life span and growth rate (Figure 8a), and the cellular morphology reverts to that seen in young normal cells (Figure 8c, d). A major downstream target of the p38 pathway is MK2, which in turn phosphorylates HSP27 leading to stress fibre formation. SB203580 treatment suppressed p38 phosphorylation, reduced the high levels of p21^WAF1^, and reduced the phosphorylated HSP27 levels inhibiting stress fibre formation (Figure 8e, f) [88,100]. Thus, SB203580 essentially prevented the accelerated ageing seen in primary WS cells. SB203580 treatment had no, or minimally, significant effects on normal cells (for effects on growth rate and lifespan see Figure 8b) [88,101]­, so this finding suggested that the abnormal growth, enlarged morphology and premature senescence of WS cultures resulted from the activation of an SB203580-suppressible pathway. 

These observations identified that small molecule treatment can largely prevent the *in vitro* ageing phenotype of cells from patients with a premature ageing syndrome, based on a variety of cellular and molecular hallmarks. The suppression of this accelerated ageing by SB203580 suggests a route whereby WS may be amenable to therapeutic intervention, and the recent development of a fully reflective mouse model of WS makes future *in vivo* studies possible to ascertain whether p38 inhibitors could be effective as therapeutic agents in a premature ageing disease [102]. Several kinase inhibitors, such as BIRB 796, VX702 and SCIO-469, which inhibit p38, proceeded into clinical trials for inflammatory conditions, including chronic diseases such as Crohn’s disease, rheumatoid arthritis, ulcerative colitis, and psoriasis, giving hope to the prospect of future clinical trials into reversing accelerated ageing [68].

### 3.2. Inhibition of MAPK Signaling in WS Cells Using Other P38 Inhibitors

The reversion of the senescent-like morphology and the greatly extended lifespan seen for WS cells treated with SB203580 suggest a potential role for both p38 and stress signaling in WS pathophysiology. If the shortened lifespan and altered cellular behaviour is reflective of *in vivo* ageing in WS individuals, this raises the possibility of this route having therapeutic potential for clinical intervention. However a therapeutic regime using SB203580 would not be suitable for *in vivo* human use due to toxicity issues [68] and the poor kinase selectivity profile of SB203580, particularly with regard to the closely related stress-associated c-Jun kinases (JNKs) and growth related kinases such as c-Raf1 [103,104], which make it difficult to draw precise conclusions, thus limiting these opportunities.

The subsequent discovery and study of many different p38 inhibitor chemotypes, such as BIRB 796 (Figure 4b), UR-13756 (Figure 4c) and RO3201195 (Figure 4d) have provided fundamental understanding of the different modes of binding and SAR that are responsible for both p38 efficacy and selectivity versus other off-target kinases, the latter of which may prove to be critical for the future design and development of effective p38 inhibitors for clinical use. Some of the p38 inhibitors, designed and tested in the on-going search for new effective treatments for certain inflammatory conditions, display similarities in cross-kinase specificity to SB203580, in particular with regard to inhibition of the stress activated c-Jun kinases (JNKs) [104,105]. Young WS cells show activation of the p46 (JNK1α1 and JNK2α1) forms of JNK, which are down-regulated by SB203580 (our unpublished data), and so facile access to a chemotype without JNK inhibitory activity is essential in order to understand the biochemical basis of why therapeutic treatment of WS cells with a p38α MAPK inhibitor rescues the phenotype.

One such MAPK inhibitor that displayed both potent activity and an exquisite selectivity profile, in particular for p38 over JNK1-3, was the Vertex Pharmaceuticals clinical candidate VX-745 (Figure 4e). This ATP-competitive p38 inhibitor exhibited the inhibition of joint degeneracy in an osteoarthritis rat model when administered twice daily at doses of up to 30 mg/kg for periods of 3 weeks [106] and clinical efficacy in rheumatoid arthritis patients using 250 mg twice daily oral administration in a 12 week randomized, double-blind, placebo-controlled trial [107,108,109]. In the latter case, VX-745 was generally well tolerated, the most significant adverse effect being an elevation of liver transaminases, which was reversible upon discontinuation of the drug. Despite its efficacy, VX-745 was withdrawn from human clinical trials due to toxicity issues, in particular the elevated liver transaminases and a mild CNS inflammation seen in a six month trial in dogs [109].

**Scheme 1:**
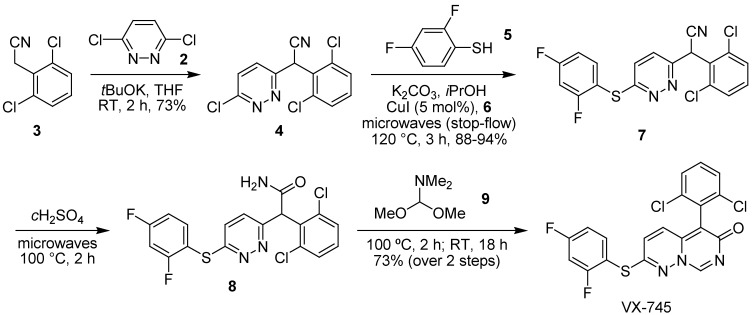
Rapid microwave-assisted synthesis of VX-745 for study in WS cells.

It has been demonstrated that rapid synthetic access to VX-745 can be achieved using a combination of conductive heating methods and microwave dielectric heating [110] to facilitate its evaluation in WS cells. The key transformation in the construction of the pyrimido[1,6-*b*]pyridazinone framework was the discovery of a microwave-mediated method for C–S bond formation that used a copper(I) catalyst and (±)-*trans*-cyclohexane-1,2-diol (**6**) ligand [111] to facilitate the synthesis of sulfide **7** from thiophenol **5** and chloropyridazine **4**, prepared in turn by chloride displacement from the reaction of dichloropyridazine **2** and acetonitrile **3**. Subsequent hydrolysis gave amide **8** which could be reacted with *N,N*-dimethylformamide dimethyl acetal (DMFDMA) (**9**) to construct the pyrimido[1,6-*b*]pyridazinone framework and deliver the target inhibitor in 50% overall yield [112] (Scheme 1). Furthermore, the Ullmann-condensation could be carried out on gramme-scale using a stop-flow microwave reactor [113], in a reproducible fashion with ready transfer of parameters, to deliver the inhibitor rapidly and efficiently and in sufficient quantities for biological evaluation. The inhibitory activity of VX-745 against p38α MAPK was confirmed in WS dermal fibroblasts at 1.0 µM concentration by immunoblot assay [112] and is 100% effective at this concentration after 24 h of pre-treatment, with an IC_50_ of approximately 50 nM [110]. In addition, VX-745 was found not to inhibit the anisomycin-induced activation of JNK1/2 or the phosphorylation of c-Jun [113], showing that, at the levels used, it was not an inhibitor of the stress-induced JNK1/2 pathway, verifying the selectivity profile of this compound and its value in the rescue of the WS phenotype. 

**Scheme 2:**
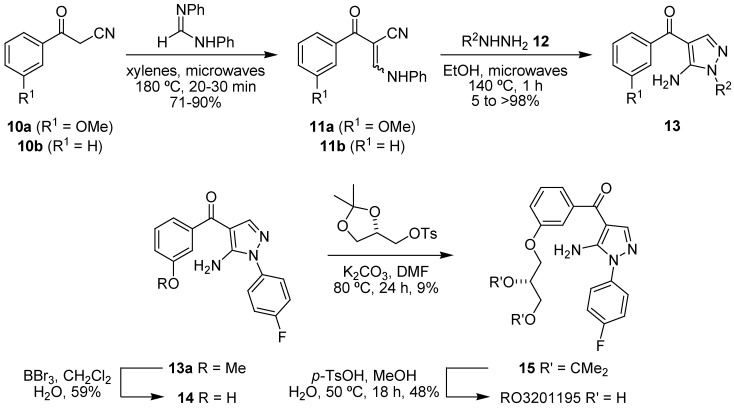
Microwave-assisted synthesis of RO3201195 and a pyrazolyl ketone library for study in WS cells.

Another chemotype of interest in the study of accelerated ageing in WS is exemplified by Roche’s 5-aminopyrazol-4-yl ketone p38 inhibitor RO3201195 (Figure 4d), which combines good cellular activity, pharmacokinetic properties, metabolic stability and bioavailability with an excellent selectivity profile as determined in a panel of 105 kinases [114]. The 5-amino-4-benzoylpyrazole motif of RO3201195 could be prepared rapidly and efficiently by a microwave-assisted route, either for elaboration to the target inhibitor [115] or in the rapid synthesis of a 24-membered pyrazolyl ketone library for the evaluation of SAR [116]. Two benzoylacetonitriles **10a,b** were reacted with *N,N′*-diphenylformamidine in a microwave-assisted Knoevenagel condensation reaction to give cyclocondensation precursors **11a,b** (Scheme 2). These β-anilino-α-benzoylacrylonitriles were then subjected to a regiospecific heterocyclocondensation by microwave irradiation with a number of hydrazines **12** to give a 24-membered 5-aminopyrazolyl ketone library **13** [116]. Further elaboration of *m*-methoxyphenyl pyrazolyl ketone **13a** to RO3201195 involved deprotection of the methyl ether by boron tribromide mediated protodemethylation, followed by *O*-alkylation of **14** with (*S*)-*O*-isopropylideneglycerol tosylate to give (*R*)-ketal **15** which could then be readily deprotected under acid-catalyzed aqueous conditions to give the (*S*)-diol RO3201195, the latter steps of which were not high yielding but did provide sufficient compound for study.

The inhibitory activity of RO3201195 prepared by this route was confirmed in hTERT-immortalized normal (HCA2) and WS dermal fibroblasts at 200 nM concentration, both by ELISA and immunoblot assay, and displayed excellent kinase selectivity for p38α MAPK over the related stress-activated kinase JNK, making it ideal for further study to rescue premature senescence and the accelerated ageing of WS cells in culture. The immuno-detection of activated versions of p38α, MK2 and HSP27 on Western blots showed that RO3201195 pre-treatment inhibited the anisomycin-induced activity of p38α, as indicated by reduced levels of activated MK2 and p-HSP27 [115]. As the level of activated MK2 was reduced in the RO3201195-treated cells compared to the control, RO3201195 was concluded to inhibit the anisomycin-induced activity of p38α *and* the p38α activity that was believed to be caused by genomic stress in the WS cells. Interestingly, RO3201195 appears to exhibit a slight increase in potency in WS cells in comparison to HCA2 cells [116]. SAR from the study of the pyrazolyl ketone library **13** indicated that large *N*-aryl groups (R^2^) were not tolerated by the p38 enzyme, in agreement with known data [114], whereas the addition of a 3-methoxy substituent in the benzoyl group caused no appreciable loss in activity. Four pyrazolyl ketones displayed activity which compared favourably with, or even exceeded, that of RO3201195, with particular promise showed by a *N*-(2,4-difluorophenyl) analogue **13b** (R^1^ = H, R^2^ = 2,4-difluorophenyl) [115].

**Figure 9:**
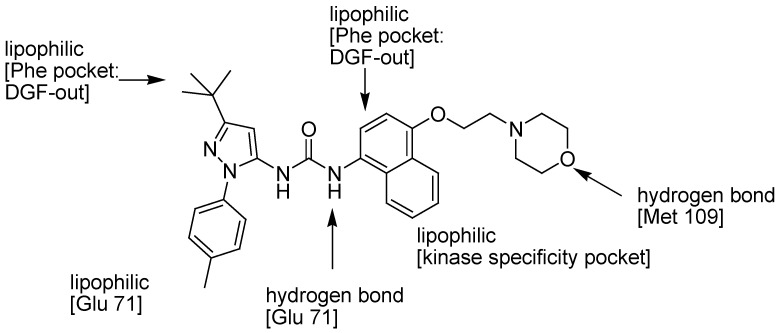
Binding mode of BIRB 796 with p38α MAPK.

A p38 inhibitor with a very different mode of action to the ATP-competitive binders is exemplified by the Boehringer Ingelheim clinical candidate BIRB 796 (Figure 4b) [105]. The class of diaryl urea inhibitors, typified by BIRB 796, utilizes an allosteric binding pocket on the kinase that is spatially distinct from the ATP pocket (Figure 9).

The formation of an allosteric binding site requires a large conformational change which is in the highly conserved Asp168-Phe169-Gly170 (DFG) motif within the active site of the kinase. In all of the known Ser/Thr kinase protein structures, the DFG motif assumes a conformation with the Phe residue buried in a hydrophobic pocket in the groove between two lobes of the kinase (DFG-in conformation) [117]. In the complex with the inhibitor, the movement of the Phe side chain to a new position (DFG-out conformation) reveals a large hydrophobic pocket in the kinase. The crystal structure of the complex between p38 MAP kinase and BIRB 796 suggests that the *t*-butyl group is inserted deep into the Phe-pocket DFG-out, which also interacts with the naphthyl ring. The tolyl substituent on the pyrazole ring has favorable interactions in a hydrophobic pocket with the Glu71 residue. This tolyl group also causes a conformational change in the Glu71 side chain inducing a hydrogen bond between only one of the urea NH groups and this residue. Moreover, the morpholino substituent makes hydrogen bond interactions with the residue of Met109 leading to affinity enhancement. This hydrogen bond, equivalent to the one made by the N1 atom of the adenine base of ATP (**1**), is crucial for the potency of the pyridinyl-imidazole inhibitors, as well as most other protein kinase inhibitors [96]. The crystal structure of human p38 MAP kinase in complex with BIRB 796 suggests that different inhibitors compete directly with the binding of ATP; others inhibit the kinase by indirectly competing with the binding of ATP. Diaryl urea inhibitors show that the DFG-out conformation is incompatible with ATP binding because the side chain of the Phe residue is in steric overlap with the phosphate groups of ATP. This class of compounds utilize binding interactions on the kinase that are spatially distinct from the adenosine 5′-triphosphate (ATP) pocket [105] stabilizing a conformation of the kinase that is incompatible with ATP binding, preventing the interaction of p38α with its upstream activating kinases MKK3 and MKK6 [118]. BIRB 796 has slow binding kinetics and so has picomolar affinity for p38α (*K_d_* 0.1 nM) and inhibits the enzyme with an IC_50_ 63 nM. The high selectivity and affinity of BIRB 796 for p38 advanced this compound into Phase III human clinical trials for the treatment of autoimmune disorders, rheumatoid arthritis and other inflammatory diseases. Characterization of BIRB 796 as a dual inhibitor of p38/JNK MAPKs has complicated interpretation of its anti-inflammatory activity [119] although the added inhibition of JNK2 has been described not to significantly contribute to the effects of BIRB796 on cytokine production. Despite this added complication, the study of this inhibitor in WS cells was still quite compelling, especially since it has been shown to inhibit all of the p38 MAPK subtypes *in vitro* and *in vivo* [120], albeit at different concentrations.

**Scheme 3:**
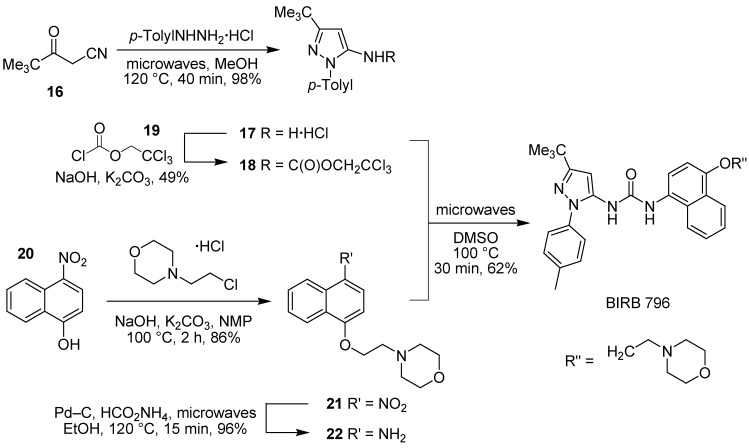
Microwave-assisted synthesis of BIRB 796 for study in WS cells.

The synthesis of BIRB 796 was found to be highly amenable to assistance using microwave dielectric heating [121] (Scheme 3). Irradiation of pivaloylacetonitrile (**16**) and *p*-tolylhydrazine hydrochloride in MeOH gave aminopyrazole **17** as its hydrochloride salt in excellent yield. Reaction with 2,2,2-trichloroethyl chloroformate (**19**) in aqueous sodium hydroxide gave carbamate **18**, which could be reacted with aminonaphthol derivative **22**, prepared by *O*-alkylation of nitronaphthol **20** followed by microwave-assisted transfer hydrogenation [122] of **21** over Pd–C in 15 min at 120 ºC. Microwave irradiation of carbamate **18** and aminonaphthol **22** in DMSO at 100 ºC gave BIRB 796 in much improved yield.

The ability of BIRB 796 to inhibit the p38α signaling pathway was tested [121] in telomerase-immortalized WS cells [88] and was shown to inhibit p38α MAPK signaling involved in the genomic stress of WS cells. BIRB 796 prevented p38α activation, with a reduction in levels of phosphorylated p38 (pp38), and pHSP27, its downstream phosphorylated target, in contrast to SB203580, which inhibited p38α activity but not its activation. Thus, BIRB 796 was confirmed as an inhibitor of the p38α signaling pathway in WS cells, thus validating its subsequent use in the study of p38α-induced accelerated ageing in this syndrome.

The pyrazolopyridine p38 inhibitor chemotype of Palau Pharma [123] has a well-defined binding mode to the ATP-binding site [124], with key interactions between substituents and a hydrophobic pocket or the hinge region in p38. From this family of compounds, the pyrazolopyridine UR-13756 (Figure 4c) was identified as a potent p38α MAPK inhibitor in cellular assays and showed good bioavailability, favorable pharmacokinetic properties, and activity in a number of inflammation models [125,126]. UR-13756 showed a much improved kinase selectivity profile over BIRB 796 and SB203580 in tests using a panel of 115 kinases, particularly in regard to the JNKs and c-Raf1 [125,126,127]. Indeed, UR-13756 even showed good selectivity for p38α over p38β, whereas SB203580 and BIRB 796 inhibit these kinases to an almost equal extent [120,127]. 

**Scheme 4:**
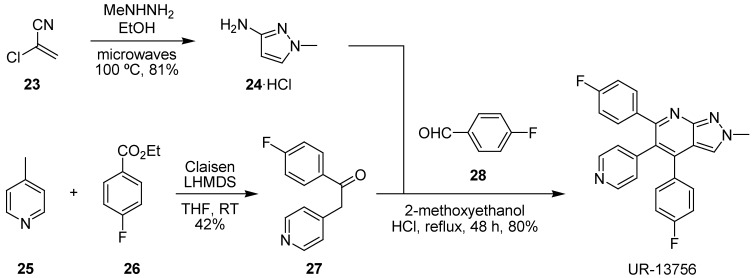
Microwave-assisted synthesis of UR-13756 for study in WS cells.

The convergent synthesis of UR-13756 was realized once again using a microwave-assisted heterocycle synthesis (Scheme 4). The pyrazole-forming reaction, in this case, was based upon heterocyclization of 2-chloroacrylonitrile (**23**) and methylhydrazine, which gave 1-methyl-3-aminopyrazole (**24**) in good yield as the only isolated regioisomer [128]. Subsequent cyclo-condensation, in a multicomponent reaction [129] related to the classical Hantzsch dihydropyridine synthesis, with aldehyde **28** and the product of a Claisen ester condensation between 4-picoline (**25**) and ethyl 4-fluorobenzoate (**26**), ketone **27**, under conductive heating conditions gave UR-13756 in gramme quantities and good overall yield suitable for evaluation in WS cells.

The inhibitory activity of UR-13756 was confirmed in hTERT-immortalized HCA2 dermal cells and WS cells by ELISA and immunoblot assay and showed excellent selectivity for p38 MAPK over JNK, as it did not prevent the anisomycin-induced phosphorylation of c-Jun, and was found to have an IC_50_ of approximately 80 nM [104]. In contrast to BIRB 796, it did not prevent the anisomycin-induced phosphorylation of p38, but prevented the phosphorylation of MK2 in WS cells. Furthermore, this inhibitor was still 100% effective at p38 inhibition in hTERT-immortalized HCA2 dermal cells after 24 h of pre-treatment at 1.0 µM.

## 4. Conclusions and Outlook

In the last ten years the discovery of the mutant gene that encodes for the WRN protein has permitted us to define the molecular pathology of Werner syndrome, a rare autosomal recessive genetic disorder exhibiting premature ageing. Primary WS cells show many of the characteristics of cells growing under ‘replication stress’, display genome instability, manifested by telomere dysfunction, an elevated rate of chromosomal translocation and genomic deletions [39] and are hypersensitive to a subset of DNA damaging agents [40]. These features suggest that the WRNp plays a key role in cellular responses to specific types of DNA damage. The genome instability seen in WS, together with frequent replication fork stalling [60], provide a plausible trigger for replication stress in WS cells and implicates the role of p38 signal transduction in their shortened replicative life span; a mechanism which we have sought to block in studies from our laboratories. Recent work [88] has identified that premature senescence probably arises from activation of the ‘stress-associated p38 mitogen activated protein kinase’, which is pivotal in orchestrating many stages of inflammation, and can be reversed using a p38 MAPK inhibitor. 

A number of different chemotypes of p38 MAPK inhibitors have been investigated in Werner syndrome cells, including the prototypical inhibitor SB203580. This cytokine-suppressive anti-inflammatory agent revealed an unexpected reversal of the WS accelerated ageing phenotype in drug-treated WS fibroblasts, which showed a much increased replicative life span and a growth rate and morphology that resembled that of normal young cells [88]. The synthesis of many other p38 inhibitor chemotypes has been carried out to enable their evaluation in WS cells in order to help rationalize the biological mechanistic basis of findings using SB203580. BIRB 796 is an allosteric inhibitor of the p38α signaling pathway and utilizes an allosteric binding pocket on p38α that is spatially distinct from the ATP pocket, stabilizing a conformation of the kinase that is incompatible with ATP binding. It is an effective inhibitor in WS cells, preventing p38α activation and reducing the levels of pp38 and pHSP27, its downstream phosphorylation target. Rapid synthetic access to VX-745 was achieved using a combination of conductive heating methods and microwave dielectric heating based upon a microwave-assisted Ullmann-condensation. Its activity was confirmed in WS dermal fibroblasts at 1.0 µM concentration with excellent selectivity over JNK. RO3201195 was prepared in a 5-step microwave-assisted linear sequence starting from a readily-available β-ketonitrile and was found to inhibit the anisomycin-induced activity of p38α and p38α activity that was believed to be caused by genomic stress in the WS cells. UR-13756 was prepared by a 3-step convergent microwave-assisted route and displayed excellent selectivity for p38 MAPK over JNK. Thus, the inhibitory profile of these chemical agents support their use as chemical tools for studying the role of p38 MAPK signaling in accelerated ageing in WS cells. These experiments are now underway in our laboratories and will be reported upon in due course.

Despite the great promise shown by SB203580 in rescuing accelerated replicative senescence in WS cells, there are still a number of issues to be addressed before there is any prospect of realizing clinical solutions to WS, even if results from screening p38 inhibitors prove to show a positive effect upon cellular growth. Firstly, the mechanistic basis of the relationship between inflammation and ageing is still not yet completely defined in Werner syndrome and so further consolidation of the biochemical signaling pathways involved remains a key goal. Furthermore, clinical trials for p38 inhibitors have identified many problems associated with their prolonged use [109]. Many p38 inhibitors are simply not efficacious *in vivo*, including Doramapimod (BIRB 796), Pamapimod, VX-702, AMG-548, Scio-323 and Scio-649, whereas others show toxicity, such as elevated liver transaminases and a mild CNS inflammation seen in a six month trial of VX-745 in dogs. Thus many, if not all, of these inhibitors are not suitable for the long-term use in humans that would be necessary for therapeutic treatment of WS. However, as inhibitors such as VX-745 and UR-13756 have proven *in vivo* efficacity [106,108,109,127] and are generally well tolerated over periods as long as 6 months in animals, they may be useful as proof-of-principle therapeutic agents in the mouse model of Werner syndrome where symptoms occur over months rather than years as is the case in humans; thus any efficacy should be seen over relatively short time scales before any serious toxicity issues occur. 

In order to progress towards a therapeutic regime, further *in vitro* studies in cultured cells are required to further elucidate the actual signaling pathways involved. Then, follow-on proof-of-principle *in vivo* studies in the Werner mouse model [130] will be required. These studies will require substantial quantities of the inhibitors so necessitating the scale up and validation of many of these routes. The scale up of microwave-assisted reactions from mg to g, or even kg, could well be required for selected p38 inhibitors (see Section 3.2) in this case. The scale up of microwave-assisted reactions is an issue that has attracted considerable attention of late and a number of different solutions are emerging [131,132,133,134]. We have presented our own resolution of these matters [135,136] and demonstrated the scale up of our synthesis of one inhibitor, VX-745, for *in vivo* study [113].

Due to the observed toxicity with p38 inhibitor use *in vivo,* a preferred option may be to target a downstream kinase. The enlarged cellular morphology with prominent F-actin stress fibres seen in young WS cells [88] suggests the involvement of the p38 downstream target MK2, as MK2 is the major kinase that phosphorylates HSP27 [137], a small heat shock protein whose activation is involved in stress fibre production. In addition, recent data suggest that MK2 acts as a checkpoint kinase that can lead to cell cycle arrest [138]. Moreover, MK2 activity up-regulates the expression of inflammatory pathways [139], and WS is associated with inflammatory diseases [5,51]. These data suggest the possibility that MK2 may be involved in the phenotypic characteristics seen in WS. If so, then therapeutic targeting of MK2 may help surmount complications in the use of p38 inhibitors, as the number of pathways inhibited will be reduced [140]. The recent disclosure of small molecule inhibitors of MK2 make this approach possible [141,142]. However, initial studies using two of these inhibitors were inconclusive as their use resulted in a complete cessation of cellular growth that appeared to be unrelated to their MK2 inhibitory action [87]. Recently, there has been a drive to develop more potent MK2 inhibitors [143,144,145], however, it is proving difficult at this time to overcome problems of selectivity and potency [146].

Overall, although progress in the development of successful p38 inhibitors for *in vivo* use is currently challenging [147], nevertheless, the study of p38 inhibition and its effect upon accelerated ageing and Werner pathophysiology is likely to provide new revelations into the biological mechanisms operating in cellular senescence and promises to link cellular signaling events to the ageing of mitotic tissues. The completion of studies of this nature may at last reveal the way forward to novel clinical solutions for the future in the treatment of Werner syndrome patients and accelerated ageing, and may thus yield important insights into the seemingly unfathomable biological mechanisms and mysteries of human ageing.
